# Structural and Functional Characterization of *Hermetia illucens* Larval Midgut

**DOI:** 10.3389/fphys.2019.00204

**Published:** 2019-03-08

**Authors:** Marco Bonelli, Daniele Bruno, Silvia Caccia, Giovanna Sgambetterra, Silvia Cappellozza, Costanza Jucker, Gianluca Tettamanti, Morena Casartelli

**Affiliations:** ^1^Department of Biosciences, University of Milan, Milan, Italy; ^2^Department of Biotechnology and Life Sciences, University of Insubria, Varese, Italy; ^3^Department of Agricultural Sciences, University of Naples Federico II, Naples, Italy; ^4^Centro di Ricerca Agricoltura e Ambiente (CREA-AA), Padua, Italy; ^5^Department of Food, Environmental and Nutritional Sciences, University of Milan, Milan, Italy

**Keywords:** bioconversion, black soldiers fly, copper cells, digestive enzymes, larval midgut, lysozyme, midgut lumen pH

## Abstract

The larvae of *Hermetia illucens* are among the most promising agents for the bioconversion of low-quality biomass, such as organic waste, into sustainable and nutritionally valuable proteins for the production of animal feed. Despite the great interest in this insect, the current literature provides information limited to the optimization of rearing methods for *H. illucens* larvae, with particular focus on their efficiency in transforming different types of waste and their nutritional composition in terms of suitability for feed production. Surprisingly, *H. illucens* biology has been neglected and a deep understanding of the morphofunctional properties of the larval midgut, the key organ that determines the extraordinary dietary plasticity of this insect, has been completely overlooked. The present study aims to fill this gap of knowledge. Our results demonstrate that the larval midgut is composed of distinct anatomical regions with different luminal pH and specific morphofunctional features. The midgut epithelium is formed by different cell types that are involved in nutrient digestion and absorption, acidification of the lumen of the middle region, endocrine regulation, and growth of the epithelium. A detailed characterization of the activity of enzymes involved in nutrient digestion and their mRNA expression levels reveals that protein, carbohydrate, and lipid digestion is associated to specific regions of this organ. Moreover, a significant lysozyme activity in the lumen of the anterior and middle regions of the midgut was detected. This enzyme, together with the strong acidic luminal pH of middle tract, may play an important role in killing pathogenic microorganisms ingested with the feeding substrate. The evidence collected led us to propose a detailed functional model of the larval midgut of *H. illucens* in which each region is characterized by peculiar features to accomplish specific functions. This platform of knowledge sets the stage for developing rearing protocols to optimize the bioconversion ability of this insect and its biotechnological applications.

## Introduction

*Hermetia illucens* (Linnaeus, 1758) (Diptera: Stratiomyidae), the black soldier fly (BSF), is a common and widespread fly in tropical and temperate regions. The insect is traditionally supposed to originate in America (Rozkošný, 1983; Wang and Shelomi, 2017) although this hypothesis was recently called into question, advancing a possible Palearctic origin (Benelli et al., 2014). Adults do not bite or sting, and have not been described as vector of any specific diseases (Wang and Shelomi, 2017). In contrast with adults, which do not need to feed according to several reports (Sheppard et al., 1994; Sheppard et al., 2002; Tomberlin and Sheppard, 2002; Tomberlin et al., 2002, 2009), the larvae of this holometabolous insect are voracious and grow on a wide variety of organic matters (Nguyen et al., 2013; Wang and Shelomi, 2017) thanks to a well-developed mandibular-maxillary complex (Kim et al., 2010).

From the pioneering study dating back to the end of 1970s (Newton et al., 1977), a growing body of evidence indicates that *H. illucens* larvae are among the most promising agents for the bioconversion of low-quality biomass (e.g., organic waste and byproducts of the agri-food transformation chain) into sustainable and nutritionally valuable proteins and lipids for the production of animal feed (Van Huis, 2013; Van Huis et al., 2013; Barragan-Fonseca et al., 2017; Wang and Shelomi, 2017). Moreover, *H. illucens* larvae are considered a potential source of bioactive substances, such as antimicrobial peptides (AMPs), a wide group of small cationic molecules that are being currently studied as a possible alternative to conventional antibiotics and food or feed preservatives (Buchon et al., 2014). The production of a wide array of AMPs by *H. illucens* could be related to the alimentary habits of the larvae, which feed on a variety of decomposing organic substrates, typically rich in microorganisms (Müller et al., 2017; Vogel et al., 2018). Furthermore, the ability of the larvae to grow on almost all organic matter makes this insect a potential source of enzymes able to degrade complex substrates that could have important industrial applications. For example, a cellulase has been characterized from *H. illucens* gut microbiota (Lee et al., 2014) and a recent review reported that BSF larvae represent a source of cellulose-, chitin-, and lignin-degrading enzymes (Müller et al., 2017). The interest in *H. illucens* as bio converter and ingredient for animal feed or source of bioactive molecules is also demonstrated by the increasing number of newly founded companies that deal with BSF mass rearing^1^.

In the last decade, despite the increasing number of publications that demonstrate the broad range of applications that can derive from the exploitation of BSF larvae, only little information on the biology of this insect has been obtained. This lack of knowledge may strongly hamper the exploitation of *H. illucens* as a source of nutrients and bioactive molecules and thus hinder any other possible future biotechnological development. Among the subjects that require more profound consideration are: characterization of the immune system, description of the gut microbiota and its relationship with the rearing substrates, a clear definition of the critical requirements for insect development, and the characterization of the morphology and physiology of the larval midgut, which has a primary role in food digestion and nutrient absorption. The latter topic, which is essential to better comprehend the extraordinary dietary plasticity of the larva and optimize the exploitation of its bioconversion capability, is the object of this study.

Although the general properties of the larval midgut of non-hematophagous Diptera belonging to the taxon of Brachycera have been already defined (Terra et al., 1996; Nation, 2008), an exhaustive and comprehensive morphofunctional characterization of this organ, with particular attention to the role and the properties of its different districts, has never been performed. Basically, the information regarding the larval midgut of Brachycera species is limited to *Musca domestica* Linnaeus, 1758 and *Drosophila* spp. In the larval midgut of *Drosophila* spp. at least three regions can be distinguished from a morphological and physiological point of view (Dimltriadis and Kastritsis, 1984; McNulty et al., 2001; Dubreuil, 2004; Shanbhag and Tripathi, 2009). Ultrastructural features of midgut cells and the mechanisms of acid and base transport across the midgut epithelium responsible for the pH values recorded in the lumen have been studied (Shanbhag and Tripathi, 2009). The anterior region is characterized by a neutral-slightly alkaline luminal pH, as is the first part of the posterior region, while the second part of the posterior region is strongly alkaline and the middle midgut is highly acidic (Shanbhag and Tripathi, 2009). A body of evidence indicates that copper cells, a peculiar cell type that is present in the first part of the middle midgut, are involved in the acidification of the lumen of this region (Dubreuil et al., 1998; McNulty et al., 2001; Dubreuil, 2004; Shanbhag and Tripathi, 2009). These cells, described for the first time by Strasburger (1932), are also known as cuprophilic or oxyntic cells.

Three regions can also be recognized in the larval midgut of *M. domestica*, characterized by a slightly acidic pH in the lumen of the anterior and posterior midgut and a strongly acidic pH in the middle region (Terra et al., 1988: Lemos and Terra, 1991a). At variance with *Drosophila* spp., the larval midgut has been functionally characterized in *M. domestica*, especially regarding the digestive properties associated to the midgut regions (Espinoza-Fuentes and Terra, 1987; Espinoza-Fuentes et al., 1987; Terra et al., 1988; Lemos and Terra, 1991b; Lemos et al., 1993; Jordão et al., 1996; Pimentel et al., 2018).

Morphofunctional studies on *H. illucens* larval midgut have never been performed. Only few data about the biochemical properties of digestive enzymes are present in the literature. Kim and coworkers (Kim et al., 2011b) investigated the enzymes released by salivary glands and gut. The authors showed that clarified gut homogenates have high amylase, lipase, and protease activities, but they did not explore whether these activities are associated to a specific region of the organ. Moreover, a qualitative and quantitative comparison of the digestive enzymatic activity from *M. domestica* and *H. illucens* larvae demonstrated that the latters possess more digestive enzymes with higher levels of activity (Kim et al., 2011b), a finding that supports the extraordinary digestive capability of *H. illucens*. Finally, two serine proteases were cloned and characterized (Kim et al., 2011a; Park et al., 2012).

The present work aims to provide an in-depth description of the morphological, ultrastructural, and functional properties of the midgut epithelium of *H. illucens* larvae, focusing attention on the peculiar characteristics of each region of this tract. In particular, we defined: (i) the morphology and ultrastructural features of the cells that form the midgut epithelium; (ii) the value of the luminal pH in the different midgut regions directing particular attention to the district where a strongly acidic pH is present; (iii) the enzymes that are involved in protein, carbohydrate, and lipid digestion, and the midgut region where they hydrolyze the substrates; and (iv) the expression levels of genes encoding for proteolytic enzymes in each midgut region. According to these data, we propose a functional model of the larval midgut of *H. illucens* that clearly shows the peculiar features and functions of each midgut region.

## Materials and Methods

### Insect Rearing

*Hermetia illucens* larvae used in this study were obtained from a colony established in 2014 at the University of Milan (Milan, Italy) starting from larvae purchased from a local dealer (Redbug, Milan, Italy).

BSF adults were kept at 27 ± 0.5°C, under a 12:12 h light:dark photoperiod, 70 ± 5% relative humidity with water supply and egg traps to promote oviposition. Eggs were collected in a Petri dish (9 cm × 1.5 cm), maintained at 27 ± 0.5°C until hatching, and then subjected to a weaning procedure as described in Pimentel et al. (2017). Lots of 300 larvae were grown on a standard diet for Diptera (Hogsette, 1992), composed of 50% wheat bran, 30% corn meal, and 20% alfalfa meal mixed at a ratio of 1:1 dry matter:water. The larvae were maintained at 27.0 ± 0.5°C, 70 ± 5% relative humidity, in the dark. Fresh diet was added to the feeding substrate every 2 days. All experiments were performed on actively feeding last instar larvae weighing between 180 and 230 mg. For Western blot analysis of phospho-Histone 3, midgut samples collected from larvae molting from third to fourth instar were also used.

### Isolation of Midgut Epithelium and Midgut Juice

**FIGURE 1:**
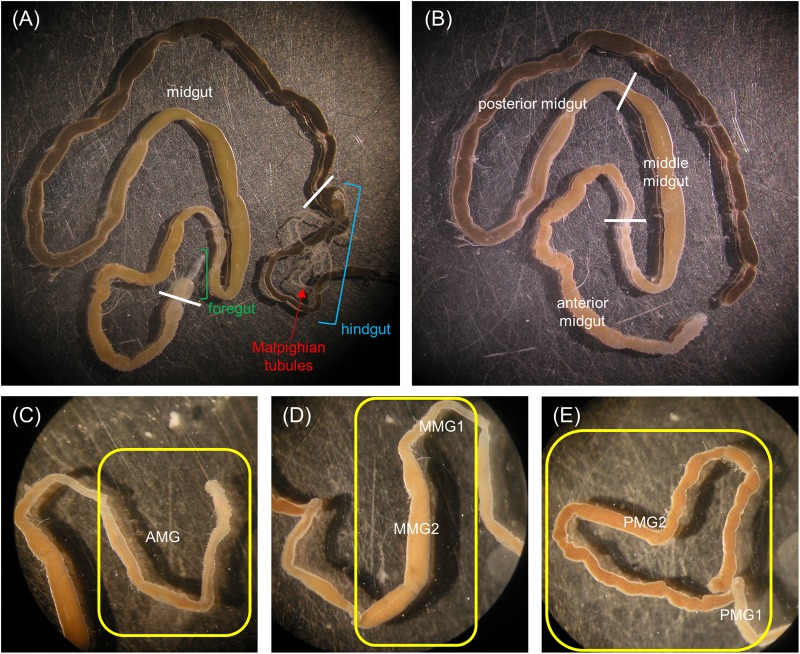
Anatomy of the alimentary canal of *H. illucens* larvae and definition of the midgut regions. The short foregut is followed by a very long midgut; the beginning of the hindgut is identifiable by the insertion of Malpighian tubules, structures involved in excretion **(A)**. The midgut can be subdivided into three main regions: anterior, middle, and posterior **(B)**. In **(C–E)** details of each midgut region are reported. Anterior midgut (AMG) **(C)**; middle midgut **(D)** in which a first narrow and short tract (MMG1) is followed by a segment with a larger diameter (MMG2); posterior midgut **(E)** in which two tracts are recognizable, a first short part (PMG1) and a second tract darker in color (PMG2).

Larvae were anesthetized on ice with CO_2_. The gut was isolated in Phosphate Buffered Saline (PBS, 137 mM NaCl, 2.7 mM KCl, 8.1 mM Na_2_HPO_4_, 1.76 mM KH_2_PO_4_, pH 7.4) at 4°C, and the foregut and the hindgut were removed. The midgut, with the enclosed intestinal content, was subdivided into five regions: anterior midgut (AMG), middle midgut 1 and 2 (MMG1 and MMG2), and posterior midgut 1 and 2 (PMG1 and PMG2) (see Figure 1 and section “General organization of the larval midgut and pH of the midgut lumen” in Results).

For morphological and immunohistochemical analyses, the five midgut regions were fixed as described in the following sections.

For the rest of the analyses, the midgut epithelium was separated from the gut content, lightly blotted on filter paper, placed into cryovials and weighed. Tissues were stored in liquid nitrogen for aminopeptidase N (APN) assay (performed on AMG, MMG2, for simplicity termed middle midgut, and PMG2, for simplicity termed posterior midgut), Western blot (performed on the whole midgut for phospho-Histone 3 and on MMG1 for H^+^ V-ATPase), and qRT-PCR analysis (performed on AMG, MMG1, MMG2, PMG1, and PMG2).

The peritrophic matrix of each midgut region, with the enclosed intestinal content, was isolated, lightly blotted on filter paper and placed into microcentrifuge tubes. Samples were then centrifuged at 15000 × *g* for 10 min at 4°C. Supernatant, i.e., the midgut juice, was collected and used immediately to measure pH value, or stored at -80°C for the enzymatic assays. For these analyses only AMG, middle midgut 2 (for simplicity termed middle midgut), and posterior midgut 2 (for simplicity termed posterior midgut) were used to avoid contaminations of midgut content between tracts.

### Light and Transmission Electron Microscopy

After isolation, midgut samples were fixed in 4% glutaraldehyde in 0.1 M Na-cacodylate buffer, pH 7.4, overnight at 4°C. After postfixation in 2% osmium tetroxide for 2 h at room temperature, specimens were dehydrated in ascending ethanol series and embedded in Epon/Araldite 812 mixture resin. Sections were obtained with a Leica Reichert Ultracut S (Leica, Nußloch, Germany). Semi-thin sections were stained with crystal violet and basic fuchsin and then observed with an Eclipse Ni-U microscope (Nikon, Tokyo, Japan) equipped with a TrueChrome II S digital camera (Tucsen Photonics, Fuzhou, China). Thin sections were stained with lead citrate and uranyl acetate and then observed with a JEM-1010 transmission electron microscope (Jeol, Tokyo, Japan) equipped with a Morada digital camera (Olympus, Münster, Germany).

### Immunohistochemistry

After isolation, midguts were fixed in 4% paraformaldehyde in PBS overnight at 4°C. Specimens were then dehydrated in ascending ethanol series and embedded in paraffin. Sections (7 μm-thick), obtained with microtome Jung Multicut 2045, were deparaffinized, rehydrated, and pre-incubated for 30 min with PBS containing 2% bovine serum albumin (BSA) before incubation with anti-H^+^ V-ATPase antibody (Ab 353-2, polyclonal antibody raised against the highly purified V_1_ complex of the H^+^ V-ATPase from *Manduca sexta* (Huss, 2001), a gift by Prof. H. Wieczorek, University of Osnabrück, Germany, dilution 1:10000 in 2% PBS/BSA), for 1 h at room temperature. After washing with PBS, sections were incubated for 1 h at room temperature with an anti-guinea pig Cy2-conjugated secondary antibody (dilution 1:200 in 2% PBS/BSA, Jackson ImmunoResearch, West Grove, PA, United States). After washes with PBS, sections were incubated with DAPI (100 ng/ml in PBS) for nuclear staining and then washed. Slides were mounted in Citifluor (Citifluor Ltd, London, United Kingdom) with coverslips and analyzed with an Eclipse Ni-U microscope (Nikon) equipped with TrueChrome II S digital camera (Tucsen Photonics). The primary antibody was omitted in controls.

### Western Blot Analysis

Tissues were homogenized with T10 basic ULTRA-TURRAX (IKA, Staufen im Breisgau, Germany) in RIPA buffer (150 mM NaCl, 2% NP-40, 0.5% sodium deoxycholate, 0.1% sodium dodecyl sulfate (SDS), and 50 mM Tris, pH 8.0) (1 ml/0.14 g of tissue), to which protease inhibitor cocktail was added (final concentrations: 1 mM AEBSF, 800 nM Aprotinin, 50 μM Bestatin, 15 μM E-64, 20 μM Leupeptin, 10 μM Pepstatin A) and phosphatase inhibitors (final concentrations: 1 mM sodium orthovanadate and 5 mM sodium fluoride) (Thermo Fisher Scientific, Waltham, MA, United States). The homogenate was centrifuged at 15000 × *g* for 15 min at 4°C and the pellet discarded. Bradford assay (Bradford, 1976) was used to determine the protein concentration of the supernatant. Clarified homogenates were denatured at 98°C in 4 × gel loading buffer for 5 min and loaded on 8% or 12% Tris-glycine acrylamide gel for SDS-PAGE analysis (60 μg protein/lane). Proteins were then transferred to a 0.45 μm nitrocellulose membrane (GE Healthcare Life Sciences, Little Chalfont, United Kingdom). The membranes were blocked with 5% milk in Tris Buffered Saline (TBS) (50 mM Tris-HCl, 150 mM NaCl, pH 7.5) for 2 h at room temperature, and subsequently incubated for 1 h at room temperature with the following antibodies: anti-phospho-Histone 3 (polyclonal antibody able to react with human and mouse phospho-Histone 3, but previously tested on different insect species (Barton et al., 2014; Apiz and Salecker, 2015; Choi et al., 2015; Franzetti et al., 2015; Franzetti et al., 2016)) (#06-570, Merck-Millipore, Burlington, MA, United States), diluted 1:1000 in TBS to which 2% milk was added, anti-H^+^ V-ATPase (Ab 353-2, see above for detail, section “Immunohistochemistry”), diluted 1:10000 in TBS to which 5% milk was added, or anti-GAPDH (Proteintech, Rosemont, IL, United States), diluted 1:2500 in TBS to which 5% milk was added. After three washes with TBS to which 0.1% Tween 20 was added, antigens of phospho-Histone 3 and GAPDH were detected with an anti-rabbit (dilution 1:7500 in TBS to which 5% milk was added; Jackson ImmunoResearch Laboratories), and antigen of H^+^ V-ATPase with an anti-guinea pig (dilution 1:10000 in TBS to which 5% milk was added, Jackson ImmunoResearch Laboratories) HRP-conjugated secondary antibodies. After three washes with TBS to which 0.1% Tween 20 was added, immunoreactivity was detected by SuperSignal chemiluminescent substrate (Thermo Fisher Scientific).

### RNA Extraction and qRT-PCR

Total RNA was extracted from 15 to 40 mg of frozen tissue by using TRIzol reagent (Thermo Fisher Scientific), according to the manufacturer’s instructions. DNA contamination was removed by using TURBO DNA-free Kit (Thermo Fisher Scientific), then the purity of RNA was assessed by quantification and the integrity of RNA was tested by electrophoresis on 1% agarose gel. RNA was retrotranscribed with M-MLV reverse transcriptase (Thermo Fisher Scientific). qRT-PCR was performed with iTaq Universal SYBR Green Supermix (Bio-Rad, Hercules, CA, United States) using a 96-well CFX Connect Real-Time PCR Detection System (Bio-Rad). To calculate the relative expression of genes of interest, the 2^-ΔΔCt^ method was used, with *HiRPL5* (*Hermetia illucens* Ribosomal Protein L5) as a housekeeping gene. The primers used for *HiTrypsin* (accession number HQ424575) were: F: ATCAAGGTCTCCCAGGTC and R: GGCAAGAGCAATAAGTTGGAT; for *HiChymotrypsin* (accession number HQ424574) were: F: AGAATGGAGGAAAGTTGGAGA and R: CAATCGGTGTAAGCAGAGACA. The primers used for *HiRPL5* (F: AGTCAGTCTTTCCCTCACGA and R: GCGTCAACTCGGATGCTA) were designed on conserved regions of *RPL5* in other insect species and the sequence was checked by sequencing the PCR product.

### Determination of Midgut Juice pH

The pH of the midgut juice isolated from midgut samples as described above was measured by universal indicator strips with a resolution of 0.5 pH unit (Hydrion Brilliant pH Dip Sticks, Sigma-Aldrich, Milan, Italy). Samples of midgut juice of the different regions were extracted from at least 15 larvae and the experiment was repeated on 14 independent batches of larvae.

### Functional Characterization of Copper Cells

Last instar larvae were transferred to standard diet prepared with a water solution containing 4 mM cupric chloride (CuCl_2_) and allowed to feed for 6, 12, 24, and 48 h. Control larvae were fed on diet prepared with water without CuCl_2_. At the indicated time points, 15 larvae were collected, anesthetized on ice with CO_2_ and dissected. The whole gut was removed and placed in ice-cold PBS. The midgut was divided into five regions as described above and fixed overnight at 4°C in 3.7% formaldehyde in PBS. Tissue samples were then rinsed in PBS and mounted on microscope slides with mounting medium (2:1 glycerol:PBS). Copper-dependent fluorescence was observed with a BX50 fluorescence microscope (Olympus) at an excitation wavelength of 365 nm, analyzing the emitted fluorescence between 585 and 620 nm.

At the same time points (6, 12, 24, and 48 h), midgut juice from 15 larvae was collected from both control and copper-fed larvae, and the luminal pH of anterior, middle, and posterior midgut region was measured as indicated above.

### Enzymatic Assays on Midgut Juice

Frozen samples of midgut juice were thawed at 4°C and protein concentration was determined by the method of Bradford (1976), using BSA as standard. Assays were performed under conditions in which product formation was linearly associated with enzyme concentration.

#### Total Proteolytic Activity

The total proteolytic activity in midgut juice samples was assayed with azocasein (Sigma-Aldrich), measuring its degradation by release of azo chromophore (Charney and Tomarelli, 1947; Vinokurov et al., 2006; Caccia et al., 2014). Different volumes of midgut juice were diluted to 100 μl with Universal Buffer (UB), which has a constant ionic strength at different pH values (Coch Frugoni, 1957); the pH used for the assays is indicated in the figure legends and in Results. Then, diluted samples were incubated for 30 min at 45°C with 200 μl of 1% (w/v) azocasein solution dissolved in UB. The reaction was terminated by adding 300 μl of 12% (w/v) trichloroacetic acid (TCA) at 4°C. The mixture was then maintained for 30 min on ice to promote undigested substrate precipitation and clarified by centrifugation at 15000 × *g* for 10 min at 4°C. An equal volume of 500 mM NaOH was added to the supernatant and absorbance was measured at 440 nm with a spectrophotometer (Pharmacia Biotech Ultrospec 3000 UV-Visible, Biochrom Ltd. Cambridge, United Kingdom). One unit (U) of total proteolytic activity was defined as the amount of enzyme that causes an increase in absorbance by 0.1 unit per min per mg of proteins. Controls were run on midgut juice samples heated to 100°C for 5 min to denature proteolytic enzymes (three replicates for each region of the midgut). The mean absorbance of controls (2.6 ± 0.5 U) was identical for the different midgut regions (unpaired *t*-test) and was subtracted from the total proteolytic activity that was measured in each experiment. The total proteolytic activity in midgut juice samples from the posterior midgut was assayed at different temperatures ranging from 10 to 70°C.

For inhibition assays of total proteolytic activity, midgut juice from the posterior region of the midgut was diluted 1:10 in UB at pH 8.5 and preincubated with 5 mM phenylmethanesulfonyl fluoride (PMSF, Sigma-Aldrich) or 0.1 mM E-64 (Sigma-Aldrich) for 15 min at 25°C. Inhibitor was omitted in controls. Total proteolytic activity in the absence and in the presence of inhibitors was measured as described above. Preliminary experiments were performed to verify the dose-dependent effect of the inhibitors on proteolytic activity, and the concentrations that guaranteed maximum inhibition were used.

#### Chymotrypsin- and Trypsin-Like Proteolytic Activity

Chymotrypsin- and trypsin-like proteolytic activity in midgut juice samples was assayed with N-succinyl-ALA-ALA-PRO-PHE-p-nitroanilide (SAAPPpNA, Sigma-Aldrich) and Na-Benzoyl-D,L-arginine 4-nitroanilide hydrochloride (BApNA, Sigma-Aldrich), respectively, measuring their degradation by release of p-nitroaniline (pNA) (Hosseininaveh et al., 2007). Different volumes of midgut juice were diluted to 300 μl with UB at pH 8.5. The diluted samples were then incubated with 300 μl of 10 mM SAAPPpNA solution dissolved in UB or with the same volume of 10 mM BApNA solution in UB obtained from a stock solution of 100 mM BApNA in dimethyl sulfoxide for 10 min at 45°C. The reaction was terminated by adding 600 μl of ice-cold 12% TCA and after 5 min at 25°C the absorbance was measured at 405 nm. One unit (U) of proteolytic activity was defined as the amount of enzyme that causes an increase in absorbance by 0.1 unit per min per mg of proteins.

Controls were run on midgut juice samples heated to 100°C for 5 min to denature proteolytic enzymes (three replicates for each region of the midgut). The mean absorbance of controls (8.3 ± 1.4 U and 3.0 ± 0.4 U for chymotrypsin- and trypsin-like proteolytic activity, respectively) was identical for the different midgut regions (unpaired *t*-test) and was subtracted from the activity measured in each experiment.

Trypsin-like activity in midgut juice samples from the posterior region was assayed at different temperatures, ranging from 10 to 70°C, at pH 8.5 to identify the optimum temperature, and at different pH values, ranging from 3.0 to 10.5, at 45°C to identify the optimum pH.

#### α-Amylase Activity

α-amylase activity in midgut juice samples was assayed using starch as substrate, measuring the amount of maltose released (Bernfeld, 1955). A standard curve was determined through linear regression of maltose absorbance at 540 nm. Different volumes of midgut juice were diluted to 595 μl in Amylase buffer (AB) (20 mM NaH_2_PO_4_, 6.7 mM NaCl, pH 6.9). Then, the diluted samples were incubated with 90 μl of 1% (w/v) soluble starch solution in AB. Controls without midgut juice sample and controls without substrate were performed for each experiment. All samples were incubated for 30 min at 45°C, and, after adding 115 μl of Color Reagent Solution (1 M sodium potassium tartrate, 48 mM 3,5-dinitrosalicylic acid, 0.4 M NaOH), were heated to 100°C for 15 min and then cooled in ice to 25°C, mixed by inversion, and their absorbance measured at 540 nm. Considering the standard curve and the absorbance in control samples, the production of maltose from enzymatic hydrolysis of starch was calculated. One unit of α-amylase activity (U) was defined as the amount of enzyme necessary to produce 1 mg of maltose per min per mg of proteins. Midgut juice samples heated to 100°C for 5 min to denature amylolytic enzymes showed no α-amylase activity. α-amylase activity in midgut juice samples from the anterior region was assayed at different pH values ranging from 3.0 to 10.5 to identify the optimum pH.

#### Lipase Activity

Lipase activity in midgut juice samples was assayed using Lipase Activity Colorimetric Assay Kit (BioVision, Milpitas, CA, United States) according to the manufacturer’s instructions.

#### Lysozyme Activity

Lysozyme activity in midgut juice samples was assayed with *Micrococcus lysodeikticus* lyophilized cells (Sigma-Aldrich), measuring the rate of lysis of bacterial cells. Different volumes of midgut juice were diluted to 20 μl with 66 mM potassium phosphate buffer, pH 6.2. Then, the diluted samples were incubated with 980 μl of a suspension of 0.02 % (w/v) *M. lysodeikticus* lyophilized cells in the same buffer. The mixture was subjected to a continuous absorbance reading at 450 nm at 45°C. One unit/ml (U/ml) of lysozyme activity was defined as the amount of enzyme that causes a decrease in absorbance by 1 unit per min per ml of midgut juice sample.

### Aminopeptidase N Activity in Midgut Homogenates

The activity of APN was assayed using L-leucine p-nitroanilide (Sigma-Aldrich) as substrate (Franzetti et al., 2015) and measuring its degradation by release of pNA. After thawing, midgut samples were homogenized with a microtube pestle in 50 mM Tris-HCl, pH 7.5 (1 ml/100 mg tissue). Different volumes of homogenate were diluted to 800 μl with the same buffer and then 200 μl of 20 mM L-leucine p-nitroanilide were added. The diluted samples were subjected to continuous absorbance readings at 410 nm at 45°C. One unit/mg (U/mg) of APN activity was defined as the amount of enzyme that releases 1 μmol of pNA per min per mg of proteins.

### Statistical Analyses

Statistical analyses were performed with R-statistical software (ver. 3.3.2). The following analyses were performed: one-way analysis of variance (ANOVA) followed by Tukey’s test, paired and unpaired *t*-tests. Statistical differences between groups were considered significant at *p*-value ≤ 0.05. The statistical analysis performed for each experiment and the *p*-values are reported in the figure legends.

## Results

### General Organization of the Larval Midgut and pH of the Midgut Lumen

Three regions could be clearly recognized in the alimentary canal isolated from *H. illucens* larvae, i.e., the foregut, the midgut, and the hindgut (Figure 1A). The organization of the gut into three regions of different embryonic origin characterizes all insects (Nation, 2008) and allows the sequential function of the gut, i.e., food ingestion, digestion and absorption of nutrients, and elimination of frass. At variance with other Brachycera (Terra et al., 1988; Dubreuil, 2004), the larval gut of *H. illucens* did not present gastric caeca (Figure 1A). The midgut, the region involved in the production and secretion of digestive enzymes and in the absorption of nutrients, represented the intermediate and the longest part of the digestive system (Figure 1B). Midgut gross morphology and pH values of the lumen content (i.e., midgut juice) revealed the presence of distinct regions in the midgut of *H. illucens* larvae. The AMG (Figure 1C), with an acidic lumen content (Table 1), was formed by a deeply infolded epithelium. The middle region was characterized by a narrow and short tract (middle midgut 1, MMG1) that continued in a segment characterized by a larger diameter (middle midgut 2, MMG2) and absence of infolding (Figure 1D); the lumen of the middle region was strongly acidic (Table 1). The posterior midgut began downstream of a constriction at the end of the middle tract and was characterized by an alkaline luminal pH (Table 1). It was the longest region of the midgut and showed a short first tract (posterior midgut 1, PMG1) followed by a second one darker in color (posterior midgut 2, PMG2) (Figure 1E).

**Table 1:** pH values in the lumen of the midgut regions of *H. illucens* larvae.

Midgut region	Mean ± SEM (n)
Anterior	5.9 ± 0.1 (14)^a^
Middle	2.1 ± 0.1 (14)^b^
Posterior	8.3 ± 0.2 (14)^c^


*Different letters indicate statistically significant differences between groups (mean ± SEM, number of replicates in parentheses. ANOVA test followed by Tukey’s test. ANOVA *p*-value < 0.001, Tukey’s test *p*-values: Middle vs. Anterior *p* < 0.001, Posterior vs. Anterior *p* < 0.001, Posterior vs. Middle *p* < 0.001).*

### Morphological Characterization of the Midgut Epithelium and Regional Differentiation

**FIGURE 2:**
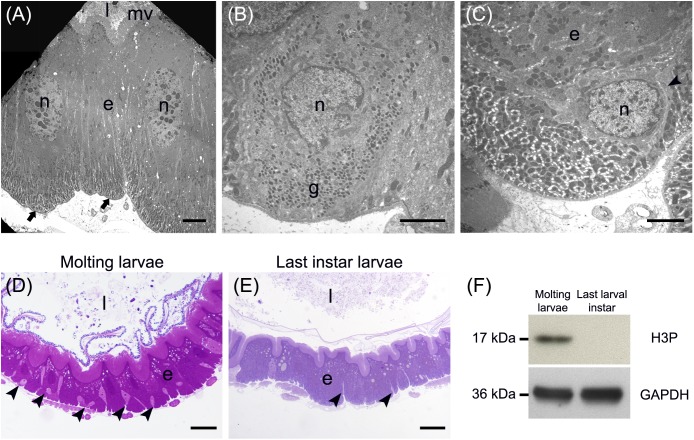
Main cell types present in the larval midgut epithelium. **(A)** Columnar cells display a developed basal infolding (arrows) and long microvilli (mv). **(B)** Endocrine cells with electron-dense granules (g) inside the cytoplasm. **(C)** Regenerative cells (arrowhead) located at the base of the midgut epithelium. **(D,E)** The number of regenerative cells (arrowheads) increases during larva-larva molt **(D)** compared to the last larval instar, when the insect is actively feeding **(E)**. **(F)** Western blot analysis of phospho-Histone 3 (H3P). e: epithelium; l: lumen; n: nucleus. Bars: 5 μm **(A)**, 2 μm **(B,C)**, 50 μm **(D)**, 20 μm **(E)**.

A detailed morphological characterization of the midgut epithelium was performed for all five regions described above (see “General organization of the larval midgut and pH of the midgut lumen” and Figure 1). Three main cell types were found along the whole length of the midgut, i.e., columnar, endocrine, and regenerative cells. Columnar cells were the most representative cell type of the epithelium and were characterized by the presence of microvilli on the apical membrane and basal infolding (Figure 2A). The endocrine cells were localized in the basal region of the epithelium and presented electron-dense granules in the cytoplasm (Figure 2B). Finally, regenerative cells were randomly distributed at the base of the epithelium (Figure 2C). These cells were able to proliferate during larva-larva molt (Figure 2D,E), as confirmed by Western blot analysis of phospho-Histone 3: a 17-kDa band (the expected molecular weight of the protein) revealed a significant presence of this mitotic marker during the molting phase, which was absent in the intermolt period (actively feeding last instar larvae) (Figure 2F and Supplementary Figure S1).

**FIGURE 3:**
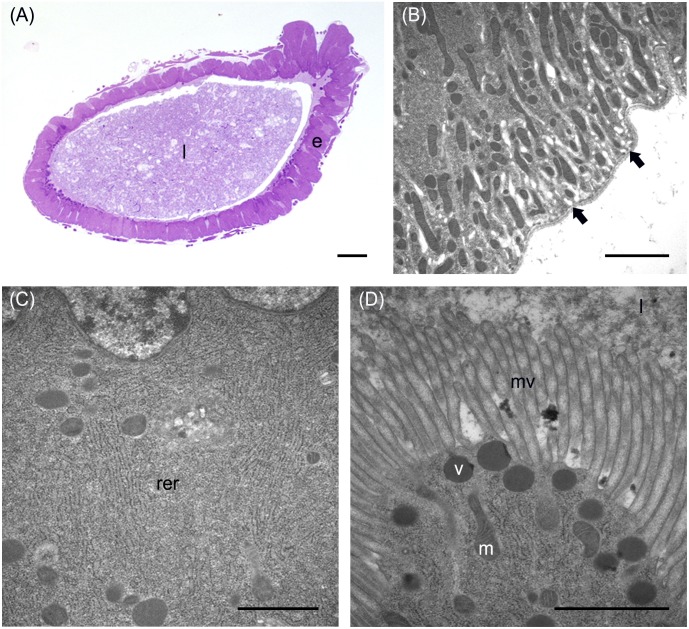
Morphological organization of the AMG. **(A)** cross-section of AMG. **(B)** Wide basal infolding (arrows) in columnar cells. **(C)** Rough endoplasmic reticulum (rer) in the cytoplasm of columnar cells. **(D)** Electron-dense vesicles (v) in the apical part of columnar cells, under the microvilli (mv). e: epithelium; l: lumen; m: mitochondria. Bars: 100 μm **(A)**, 2 μm **(B)**, 1 μm **(C,D)**.

**FIGURE 4:**
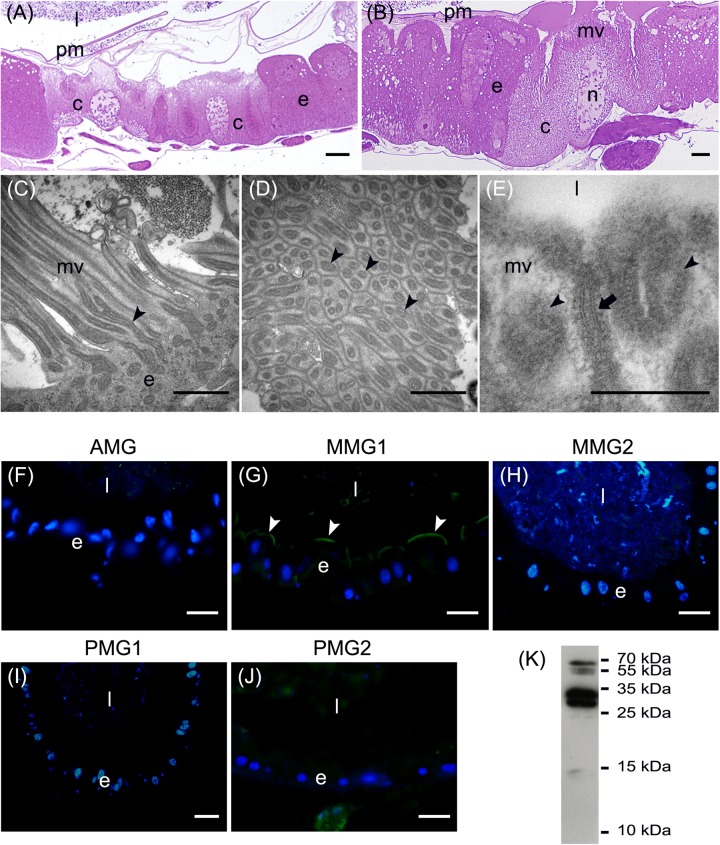
Morphological organization of MMG1. **(A,B)** Copper cells (c) characterized by the long microvilli (mv). **(C,D)** Elongated mitochondria (arrowheads) are visible in longitudinal **(C)** and cross **(D)** section of microvilli (mv). **(E)** Portasome-like structure (arrow) in the apical surface of microvilli. Mitochondria (arrowheads) are visible. **(F–J)** H^+^ V-ATPase immunolocalization (white arrowheads) in the different midgut regions. **(K)** Western blot analysis of H^+^ V-ATPase. e: epithelium; l: lumen; n: nucleus; pm: peritrophic matrix. Bars: 20 μm **(A,G–K)**, 10 μm **(B)**, 1 μm **(C,D)**, 200 nm **(E)**.

**FIGURE 5:**
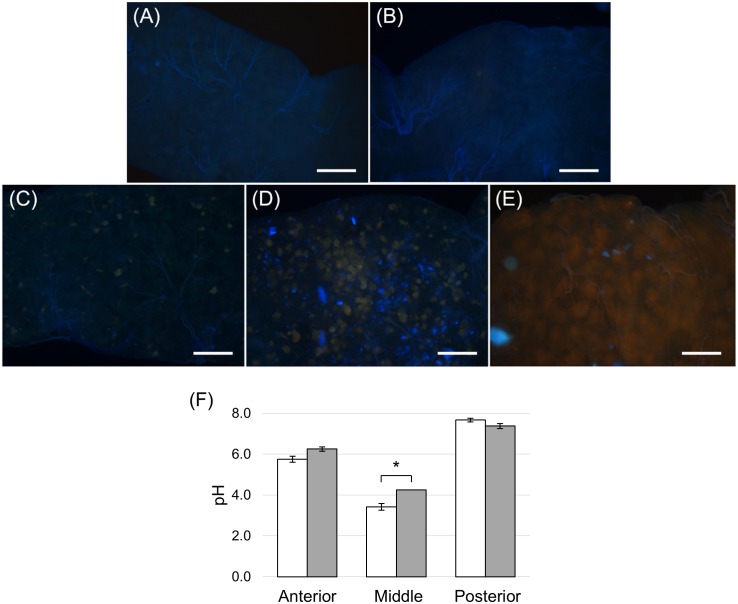
Copper feeding experiments. **(A)** Dissected midgut from control larvae reared on standard diet without CuCl_2_ for 24 h. **(B–E)**: dissected midgut from larvae reared on standard diet added with CuCl_2_ for 6, 14, 24 and 48 h, respectively. **(F)** pH measurement of midgut juice samples from anterior, middle, and posterior regions of the midgut isolated from larvae fed for 24 h on control (white bar chart) and copper-containing diet (gray bar chart). The values are reported as mean ± SEM of at least 3 experiments. Only in the middle midgut a significant difference between groups was observed (unpaired *t*-test: ^∗^*p* < 0.01). Bars: 100 μm **(A–E)**.

In addition to the three cell types described above, every midgut district presented typical features, indicating a marked regional differentiation of this organ. The AMG showed a thick epithelium (Figure 3A) formed by columnar cells with a developed basal infolding (Figure 3B). These cells were characterized by abundant rough endoplasmic reticulum (RER) (Figure 3C), and numerous electron-dense granules and mitochondria were present in the apical region under the microvilli (Figure 3D). The epithelium of MMG1 contained specialized cells, i.e., copper cells, characterized by a cup shape (Figure 4A), a large central nucleus (Figure 4B), and long microvilli (Figure 4B). A peculiar trait of these cells was the presence of several and elongated mitochondria inside each microvillus (Figure 4C,D). On the apical membrane of the cells portasome-like particles could be observed (Figure 4E). The presence of H^+^ V-ATPase, previously shown to be associated to portasomes (Shanbhag and Tripathi, 2009; Zhuang et al., 1999), was investigated by immunohistochemistry and Western blot analysis. Immunostaining performed on different midgut regions confirmed the presence of H^+^ V-ATPase only in MMG1, specifically associated with the apical membrane of copper cells (Figure 4F–J). To confirm that the antibody specifically recognized H^+^ V-ATPase in this midgut district, MMG1 was isolated and analyzed by Western blotting. Seven bands with a molecular mass of 13, 14, 27, 34, 55, 56, and 67-kDa, corresponding to most of the subunits of the cytoplasmic V_1_ complex of H^+^ V-ATPase, were recognized by the antibody (Figure 4K). The ability of these cells to acidify the middle midgut lumen, thanks to the secretion of proton into the lumen *via* H^+^ V-ATPase, was evaluated. In copper-fed larvae of *Drosophila melanogaster* Meigen, 1830, copper cells have been shown to acquire an orange fluorescence signal due to the formation of a copper ions-metallothionein complex and, in turn, the acid-secreting activity of these cells is reduced (McNulty et al., 2001). To verify whether copper cells in *H. illucens* midgut showed similar features, larvae fed on diet containing CuCl_2_ for different time periods (6, 14, 24, and 48 h) were examined. MMG1, the region containing copper cells, from control larvae reared without CuCl_2_ in the diet did not exhibit orange fluorescence when examined under UV excitation for any of the time periods considered (Figure 5A and data not shown). Conversely, the same midgut tract of larvae fed on copper-containing diet showed a fluorescence signal starting from 14 h that increased significantly over time (Figure 5B–E). Midgut cells of the other regions showed an orange fluorescence only in larvae fed on copper-containing diet for 48 h (data not shown). We also examined the relationship between copper-dependent fluorescence and midgut lumen pH. Midgut juice samples from anterior, middle, and posterior region of the midgut were extracted from larvae fed for 24 h with copper-containing diet and control diet, and the pH was measured using universal indicator strips. As indicated in Figure 5F, copper feeding reduced larval midgut acidification in the middle midgut, whereas no variation was recorded in the anterior and posterior midgut.

**FIGURE 6:**
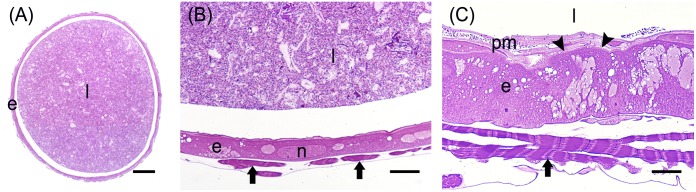
Morphological organization of MMG2. **(A)** Cross-section of MMG2. **(B,C)** Details of the large flat cells that forms the thin epithelium in this midgut region. The short brush border (arrowheads) is visible in **(C)**. e: epithelium; l: lumen; arrows: muscle cells; n: nucleus; pm: peritrophic matrix. Bars: 200 μm **(A)**, 20 μm **(B)**, 10 μm **(C)**.

MMG2 showed a wide lumen surrounded by a thin epithelium (Figure 6A) formed by large flat cells with a large elongated nucleus (Figure 6B) and a very short brush border (Figure 6C).

**FIGURE 7:**
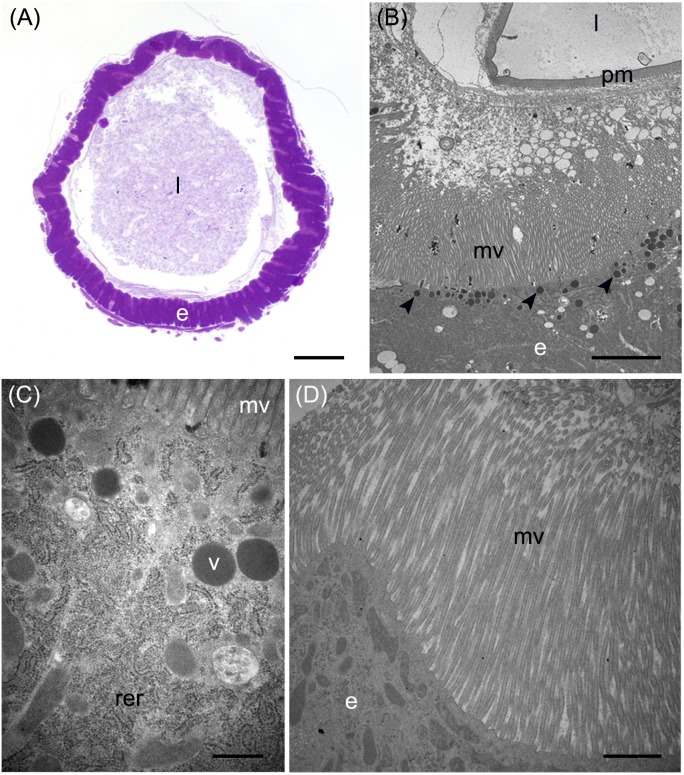
Morphological organization of the posterior midgut (PMG1 and PMG2). **(A)** Cross-section of PMG1. **(B,C)**: rough endoplasmic reticulum (rer), microvilli (mv), and electron-dense vesicles (arrowheads in **B**, v in **C**) in the apical part of columnar cells in PMG1. **(D)** Microvilli (mv) of the epithelial cells in PMG2. e: epithelium; l: lumen; pm: peritrophic matrix. Bars: 100 μm **(A)**, 5 μm **(B)**, 500 nm **(C)**, 1 μm **(D)**.

The posterior midgut displayed peculiar morphological features. In this region, the epithelium was thick (Figure 7A) with a well-developed brush border (Figure 7B). In particular, columnar cells in PMG1 differed from those of PMG2 for the numerous electron-dense granules localized under the microvilli (Figure 7B). Similar to the AMG, abundant RER (Figure 7C) was observed. Columnar cells of PMG2 showed very long microvilli (Figure 7D).

### Distribution of Digestive Enzymes in the Midgut

A previous study indicated that salivary glands of *H. illucens* larvae are only a minor source of digestive enzymes and that digestion is mainly accomplished by the midgut (Kim et al., 2011b). To evaluate whether the morphological differences observed in the different midgut regions as well as the strong variations in luminal pH along this organ corresponded to a functional regionalization, we examined the activity of enzymes involved in protein and carbohydrate digestion in each midgut region.

**FIGURE 8:**
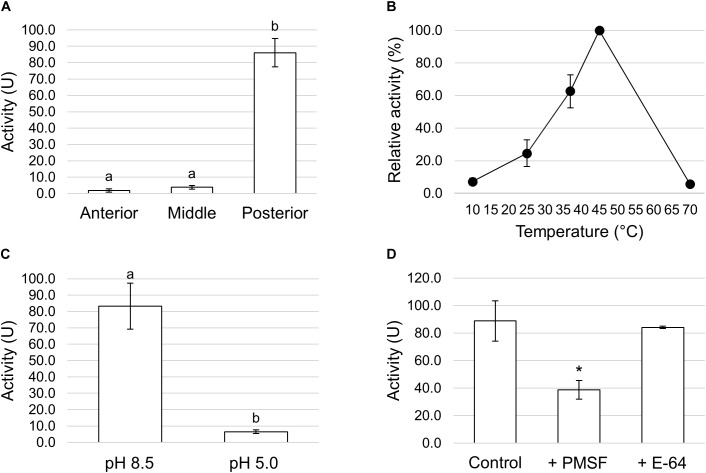
Total proteolytic activity in the different midgut regions. **(A)** Total proteolytic activity in midgut juice extracted from anterior, middle, and posterior midgut. For each tract the enzymatic assay was performed at a pH value as close as possible to that of the lumen (pH 6.0 for the AMG, pH 5.0 for the middle midgut, and pH 8.5 for posterior midgut). The values are reported as mean ± SEM of at least 3 experiments. Different letters denote significant differences (ANOVA test followed by Tukey’s test. ANOVA *p*-value < 0.001, Tukey’s test *p*-values: Middle vs. Anterior *p* = 0.975, Posterior vs. Anterior *p* < 0.001, Posterior vs. Middle *p* < 0.001). **(B)** Dependence of total proteolytic activity on temperature in midgut juice from the posterior region of the midgut performed at pH 8.5. Relative activity values (%) are reported as mean ± SEM of at least 3 experiments and are expressed as percentage of the highest activity over the temperature range examined. **(C)** Total proteolytic activity in midgut juice from the posterior midgut measured at pH 8.5 and 5.0. The values are reported as mean ± SEM of 3 experiments. Different letters denote significant differences (paired *t*-test: *p*-value < 0.05). **(D)** Total proteolytic activity in midgut juice from the posterior midgut measured at pH 8.5 in the absence (control) and in the presence of serine- (PMSF) and cysteine- (E-64) protease inhibitors. The values are reported as mean ± SEM of 5 experiments. A significant difference between groups was observed for the activity measured in the presence of PMSF vs. control (paired *t*-test: ^∗^*p*-value < 0.01).

We first measured the total proteolytic activity in the midgut juice isolated from the anterior, middle, and posterior regions using azocasein as substrate at pH values as close as possible to those present in the lumen of each region. As reported in Figure 8A, the posterior tract showed the highest activity, which was more than forty- and twenty-fold higher than in the anterior and middle region, respectively. Since the posterior midgut may have a major role in protein digestion, a detailed characterization of the total proteolytic activity in this region was performed. First, we evaluated the dependence of the total proteolytic activity on temperature (Figure 8B). The highest activity was recorded at 45°C, while beyond this temperature the residual activity declined, reaching approximately 10% at 10 and 70°C. Since the lumen of the posterior midgut had an alkaline pH (Table 1), we wanted to determine whether the total proteolytic activity measured in this region could be ascribed to serine proteases, the major family of endopeptidases that shows a significant activity at alkaline pH values (Terra and Ferreira, 1994, 2005). When the proteolytic activity was measured at acidic pH (pH 5.0), a highly significant decrease in the enzymatic activity (approximately 13-fold reduction) was observed compared to that recorded at pH 8.5 (Figure 8C). Moreover, PMSF, a rather specific, competitive, and irreversible inhibitor of serine proteases, caused a significant reduction in the total proteolytic activity compared to controls, with an inhibition of 56.5 ± 1.4% (Figure 8D). In contrast, no inhibition was observed with E-64, an irreversible inhibitor of a wide range of cysteine proteases (Figure 8D).

**FIGURE 9:**
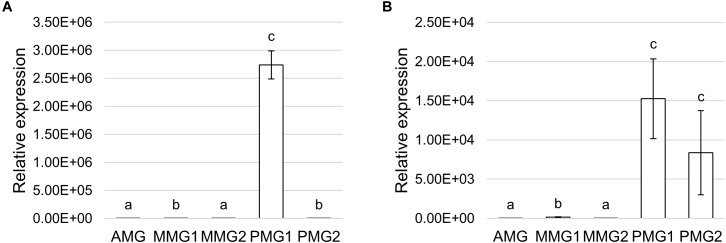
Expression profile of serine proteases. **(A,B)** qRT-PCR analysis of *HiTrypsin* **(A)** and *HiChymotrypsin* **(B)** in the different midgut regions. The values are reported as relative expression mean ± SEM of 3 experiments. Different letters denote significant differences: *HiTrypsin* (ANOVA test followed by Tukey’s test. ANOVA *p*-value < 0.001, Tukey’s test *p*-values: AMG vs. MMG1 *p* < 0.001, AMG vs. MMG2 *p* = 0.107, AMG vs. PMG1 *p* < 0.001, AMG vs. PMG2 *p* < 0.001, MMG1 vs. MMG2 *p* < 0.05, MMG1 vs. PMG1 *p* < 0.001, MMG1 vs. PMG2 *p* = 0.328, MMG2 vs. PMG1 *p* < 0.001, MMG2 vs. PMG2 *p* < 0.001, PMG1 vs. PMG2 *p* < 0.001); *HiChymotrypsin* (ANOVA test followed by Tukey’s test. ANOVA *p*-value < 0.001, Tukey’s test *p*-values: AMG vs. MMG1 *p* < 0.01, AMG vs. MMG2 *p* = 0.237, AMG vs. PMG1 *p* < 0.001, AMG vs. PMG2 *p* < 0.001, MMG1 vs. MMG2 *p* < 0.001, MMG1 vs. PMG1 *p* < 0.01, MMG1 vs. PMG2 *p* < 0.01, MMG2 vs. PMG1 *p* < 0.001, MMG2 vs. PMG2 *p* < 0.001, and PMG1 vs. PMG2 *p* < 0.790).

**FIGURE 10:**
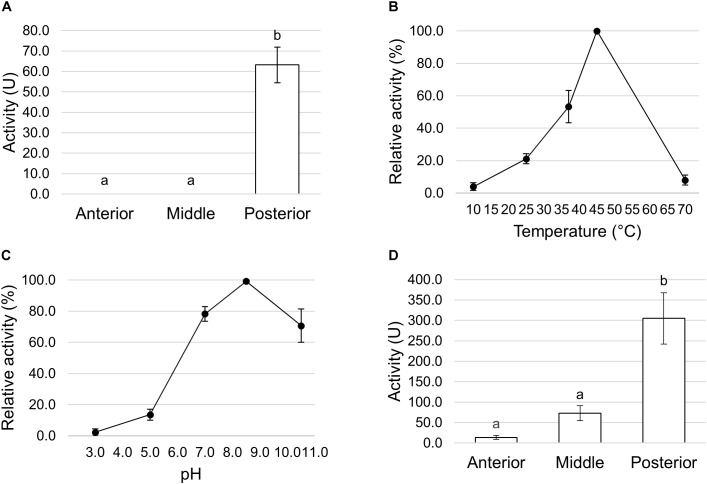
Trypsin and chymotrypsin activity in the different midgut regions. **(A)** Trypsin activity in midgut juice extracted from anterior, middle, and posterior midgut. For each tract the enzymatic assay was performed at pH 8.5. The values are reported as mean ± SEM of 3 experiments. Different letters denote significant differences (ANOVA test followed by Tukey’s test. ANOVA *p*-value < 0.001, Tukey’s test *p*-values: Middle vs. Anterior *p* = 1.000, Posterior vs. Anterior *p* < 0.001, Posterior vs. Middle *p* < 0.001). **(B)** Dependence of trypsin activity on temperature in midgut juice from the posterior region of the midgut performed at pH 8.5. Relative activity values (%) are reported as mean ± SEM of at least 3 experiments and are expressed as percentage of the highest activity over the temperature range examined. **(C)** Dependence of trypsin activity on pH in midgut juice from the posterior region of the midgut performed at 45°C. Relative activity values (%) are reported as mean ± SEM of at least 3 experiments and are expressed as percentage of the highest activity over the pH range examined. **(D)** Chymotrypsin activity in midgut juice extracted from anterior, middle, and posterior midgut. For each tract the enzymatic assay was performed at pH 8.5. The values are reported as mean ± SEM of at least 3 experiments. Different letters denote significant differences (ANOVA test followed by Tukey’s test. ANOVA *p*-value < 0.05, Tukey’s test *p*-values: Middle vs. Anterior *p* = 0.845, Posterior vs. Anterior *p* < 0.05, Posterior vs. Middle *p* = 0.061).

Trypsin- and chymotrypsin-like enzymes are the major serine proteases involved in protein digestion in most insects (Terra and Ferreira, 1994, 2005). To verify the involvement of these enzymes in digestion processes, we first measured mRNA levels of two isoforms of these enzymes (Kim et al., 2011a) in the different midgut districts by qRT-PCR. Both genes were highly expressed in the posterior midgut, mRNA levels being significantly higher in PMG1 than in PMG2 for *HiTrypsin* (Figure 9A,B). By using specific substrates, we measured the tryptic and chymotryptic activity in the different midgut regions. Figure 10A reports trypsin-like activity in the midgut juice from anterior, middle, and posterior region of the midgut using BApNA as substrate. No activity was recorded in the anterior and middle region, while a significant trypsin-like activity was present in the posterior region. To better characterize the tryptic activity, we evaluated its dependence on temperature and pH. The optimal temperature, as for the total proteolytic activity (Figure 8B), was 45°C (Figure 10B). The evaluation of pH effect on trypsin activity at a temperature of 45°C showed that the highest BApNA hydrolysis occurred at pH values from 7.0 to 10.5, with an optimum at pH 8.5, and decreased significantly at acidic pH (Figure 10C). We also measured the activity of chymotrypsin-like proteases in the midgut juice extracted from the three regions of the midgut using SAAPPpNA as substrate (Figure 10D). The highest activity was recorded in the lumen content of the posterior midgut, confirming that this district is the main site for protein digestion by endopeptidases belonging to the serine proteases family.

**FIGURE 11:**
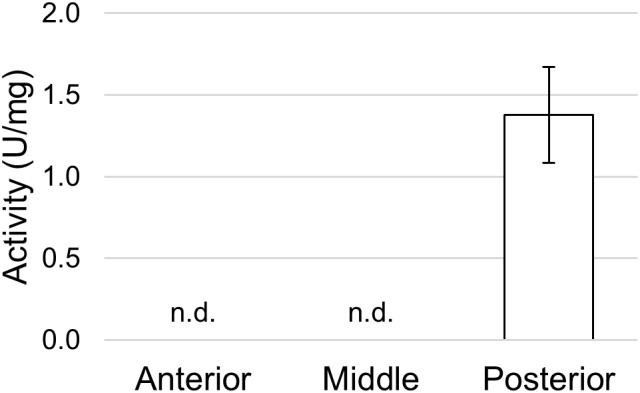
Aminopeptidase N activity in the different midgut regions using L-leucine p-nitroanilide as substrate. The values are reported as mean ± SEM of at least 3 experiments. In the anterior and middle midgut no activity was detected (n.d.).

To define whether the posterior midgut was also responsible for the final phase of protein digestion in which single amino acids are produced, we measured the activity of APN, one of the abundant exopeptidase families present in the midgut brush border of insect larvae (Terra and Ferreira, 1994). APN activity was recorded only in tissue homogenates prepared from the posterior midgut (Figure 11).

**FIGURE 12:**
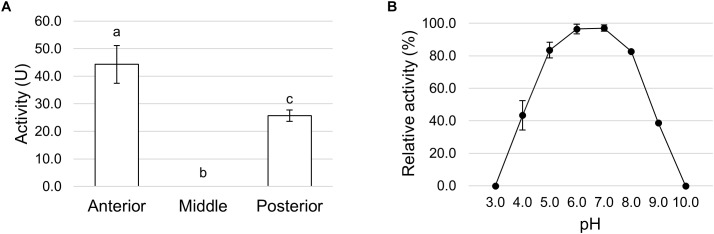
α-amylase activity in the different midgut regions. **(A)** α-amylase activity in midgut juice extracted from anterior, middle, and posterior midgut. The values are reported as mean ± SEM of at least 3 experiments. Different letters denote significant differences (ANOVA test followed by Tukey’s test. ANOVA *p*-value < 0.001, Tukey’s test *p*-values: Middle vs. Anterior *p* < 0.001, Posterior vs. Anterior *p* < 0.05, Posterior vs. Middle *p* < 0.05). **(B)** Dependence of α-amylase activity on pH in midgut juice from the anterior region of the midgut. Relative activity values (%) are reported as mean ± SEM of at least 3 experiments and are expressed as percentage of the highest activity over the pH range examined.

α-amylase activity in midgut juice samples from anterior, middle, and posterior regions of the midgut was also determined. The highest activity was recorded in the AMG, although starch was also digested to a significant degree in the posterior region; conversely, no activity was present in the middle midgut (Figure 12A). As the pH of the lumen in the three midgut regions was rather different in *H. illucens* larvae (Table 1), we performed a detailed evaluation of pH dependence in the AMG to assess how this chemical parameter influenced α-amylase activity. High activity was recorded at pH values ranging from 5.0 to 8.0, with a maximum between pH 6.0 and 7.0. At pH values lower than 5.0 and higher than 8.0 α-amylase activity significantly decreased and dropped to zero at pH 3.0 and 10.0 (Figure 12B). Therefore, the luminal pH value of the anterior and posterior midgut (Table 1), the regions where hydrolysis of internal α-1,4-glycosidic bonds of polysaccharides (i.e., starch and glycogen) occurs (Figure 12A), fits with the optimum pH range of α-amylase activity (Figure 12B).

We also measured lipase activity in midgut juice samples from anterior, middle, and posterior regions of the midgut. A significant activity was recorded in the anterior (4.51 ± 0.24 U/mg, mean ± SEM of 4 experiments) and in the posterior midgut (6.86 ± 0.62 U/mg, mean ± SEM of 6 experiments), in contrast, no lipase activity was detected in the middle region (Supplementary Figure S2).

**FIGURE 13:**
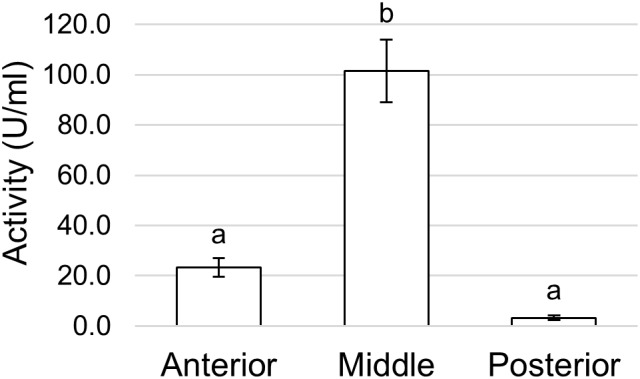
Lysozyme activity assayed in the different midgut regions measuring the rate of lysis of *Micrococcus lysodeikticus*. The values are reported as mean ± SEM of at least 3 experiments. Different letters denote significant differences (ANOVA test followed by Tukey’s test. ANOVA *p*-value < 0.001, Tukey’s test *p*-values: Middle vs. Anterior *p* < 0.001, Posterior vs. Anterior *p* = 0.394, Posterior vs. Middle *p* < 0.001).

Finally, since the midgut can be involved in the enzymatic clearance of ingested microorganisms (Lemos and Terra, 1991a; Lemos et al., 1993; Terra and Ferreira, 1994; Padilha et al., 2009), we measured the activity of lysozyme, which catalyzes the hydrolysis of 1,4-β-glycosidic bonds between N-acetylmuramic acid and N-acetyl-D-glucosamine residues in the peptidoglycan of the cell wall of many bacteria, thus compromising the integrity of the structure and causing the lysis of the cell. Lysozyme activity was measured in midgut juice samples extracted from anterior, middle, and posterior regions of the midgut. The highest activity was present in the middle midgut, whereas the other two regions showed significantly lower activities, especially the posterior region (Figure 13).

## Discussion

The great interest in *H. illucens* larvae for their ability to bioconvert low quality substrates into valuable biomass (Barragan-Fonseca et al., 2017; Müller et al., 2017; Wang and Shelomi, 2017) is not supported by knowledge of the physiology of this insect, in particular of the functional properties of the midgut, which is involved in nutrient digestion and absorption. This lack of information can negatively affect exploitation of the bioconversion ability of *H. illucens* as the dietary plasticity of the larvae and the efficiency of bioconversion strictly depend on the physiological properties of this organ. In this work, we have filled this gap of knowledge by performing an in-depth morphofunctional characterization of the larval midgut.

Our results indicate that the midgut of *H. illucens* larvae shows a marked regionalization and each region possesses peculiar morphological and functional features. A key parameter that confirms midgut regionalization is the pH of the lumen. In fact, the three main regions of the midgut present very different pH values, being acidic, strongly acidic and alkaline in the anterior, middle, and posterior midgut, respectively. Although the pH values of the two first regions are similar to those measured in other Brachycera larvae (Terra et al., 1988; Shanbhag and Tripathi, 2009), the alkaline pH in the posterior midgut is not a common feature: in fact, although in *D. melanogaster* the pH of the lumen in this region is higher than 10 (Shanbhag and Tripathi, 2009), a slightly acidic pH (pH 6.8) was measured in *M. domestica* (Terra et al., 1988). It should be highlighted that luminal pH plays a crucial role in the functional properties of the midgut since it influences digestive enzyme activity, solubility of nutrients, neutralization of toxic ingested compounds, and gut microbiota (Appel, 1994; Nation, 2008; Bruno et al., 2019).

Our data show that the epithelium of the AMG of *H. illucens* larvae is characterized by the presence of columnar cells that possess structural and ultrastructural properties typical of secretory cells and are likely responsible for the production and secretion of amylases, lipases, and lysozyme, whose activity has been recorded in the lumen of this region. Ingested polysaccharides begin to be degraded in the AMG by soluble amylases that show the highest activity in this tract. In other brachycerous larvae, such as *M. domestica*, amylase expression (Pimentel et al., 2018) and activity (Espinoza-Fuentes and Terra, 1987) have been recorded especially in the posterior midgut, as for the majority of digestive enzymes (Espinoza-Fuentes and Terra, 1987). It is difficult to explain the differing localizations of carbohydrate digestion in the two species on the basis of their alimentary habits because both insect larvae are saprophagous and feed on very similar substrates, thus, the explanation might possibly be related to the phylogenetic distance between the two species (Wiegmann et al., 2011). The AMG, together with the posterior tract, is also responsible for lipid digestion. Although lipids are important components of insect diets, only few studies about lipid digestion in insects are available and on only little is known about the biochemical features of lipases (Canavoso et al., 2004; Grillo et al., 2007; Zibaee et al., 2008; Zibaee, 2012; Weidlich and Hoffmann, 2015; Santana et al., 2017).

The first part of the middle midgut (MMG1) is characterized by the presence of copper cells. ATP produced by the elongated mitochondria inside microvilli is readily available to be used by H^+^ V-ATPase localized in the apical membrane of these cells. By Western blot analysis multiple bands that correspond to the different subunits of the V_1_ complex were revealed. In particular, the 67- and 56-kDa bands correspond to the A and the B subunits, respectively, which are responsible for the binding of ATP (Novak et al., 1992; Wieczorek et al., 1999). The remaining bands correspond to other V_1_ subunits, such as E (27-kDa) (Gräf et al., 1994a), F (14-kDa), G (13-kDa) (Gräf et al., 1994b, 1996; Lepier et al., 1996), H (55-kDa), and D (32-kDa) (Merzendorfer et al., 2000). Our data and evidence reported in the literature for other brachycerous larvae (Terra and Ferreira, 1994; Dubreuil et al., 1998; Dubreuil, 2004) indicate that copper cells, thanks to their ability to secrete H^+^, are responsible for the acidification of middle midgut lumen. In fact, as demonstrated in *D. melanogaster* larvae (McNulty et al., 2001), when this ability is impaired by copper ingestion and the metal selectively accumulates in these cells, the pH value in the middle midgut increases. As previously suggested, the orange fluorescence signal is probably due to the formation of a complex between copper ions and a protein belonging to the metallothionein family (McNulty et al., 2001). It has been demonstrated that two metallothionein genes are constitutively expressed in the middle midgut of *D. melanogaster* larvae and adult, whereas are inducible in other regions of the gut (Durliat et al., 1995). A similar pattern can also occur in *H. illucens* larvae, as the fluorescence signal appears more rapidly in copper cells (starting from 14 h of feeding on copper-containing diet) than in cells of other midgut regions, where the signal is visible only after 48 h. The physiological significance and the relationship between florescence due to formation of the complex copper-metallothionein and acid secretion are not clear. Different hypotheses have been proposed (McNulty et al., 2001), but we can exclude that the reduction of middle midgut acidification by copper is due to non-specific and detrimental effects of the metal on midgut cells, as the anterior and the posterior regions maintained their ability to regulate their own lumen pH. Another key factor essential for the acidification of the lumen of the middle midgut may be carbonic anhydrase, because this enzyme generates H^+^ ions that are transported into the lumen (Shanbhag and Tripathi, 2008, 2009).

The epithelium in the second part of the middle midgut (MMG2) shows a peculiar morphology as it is formed by large, flat cells. This region does not appear to be involved in digestive processes, but only lysozyme activity has been recorded. The high activity of this enzyme in the middle midgut, together with the strong acidic luminal pH, suggests an important role of this region in killing pathogens ingested with the feeding substrate, as proposed for other brachycerous larvae (Lemos and Terra, 1991a; Lemos et al., 1993; Padilha et al., 2009). Recently, lysozyme and extreme pH values in the middle midgut of *H. illucens* larvae have been suggested to be among those agents possibly responsible for shaping the microbiota composition of the midgut and the microbial density along the different regions of this organ (Bruno et al., 2019).

Our data indicate that the posterior midgut plays a fundamental role in protein digestion. In this region, endo- and exo-peptidases accomplish the hydrolysis of peptide bonds that leads to the production of free amino acids. Columnar cells are the main cell type present in the posterior region, but they show different morphological features in the first and in the second part of this district that confer different functional properties to these two tracts. Columnar cells present in the first part of the posterior midgut (PMG1) are likely characterized by secretory activity. This evidence is supported by qRT-PCR data that demonstrate a primary involvement of PMG1 in the production of serine proteases, i.e., trypsin and chymotrypsin. These two enzymes are responsible for the initial phase of protein digestion that occurs in the posterior midgut. Moreover, as the assay conditions for the measurement of trypsin and chymotrypsin activity are the same and both specific chromogenic substrates release p-nitroaniline after the hydrolysis, it is possible to compare the relative activity of the two enzymes. Considering their activity in the posterior region of the midgut, chymotrypsin-like enzymes appear to be the major serine proteases responsible for the initial phase of protein digestion. In the second part of the posterior midgut (PMG2), columnar cells have microvilli that are longer than in other regions while morphological traits ascribable to a secretory activity are less evident, supporting a main role of this tract in nutrient absorption. Moreover, APN activity is recorded only in the posterior region, confirming that this district plays a fundamental role in protein digestion from the initial to the final phases of the process, the latter producing single amino acids that, in turn, can be absorbed. *H. illucens* larvae are able to grow on a great variety of organic matter (Nguyen et al., 2013; Barragan-Fonseca et al., 2017; Wang and Shelomi, 2017), including vegetable materials (Jucker et al., 2017), a substrate rich in tannins and other secondary metabolites which bind to proteins at low pH values, affecting the efficiency of digestion (Felton et al., 1989; Felton and Duffey, 1991; Appel, 1994). Considering this evidence, the digestion of proteins in the posterior midgut, a region with an alkaline pH of lumen, could ensure the best exploitation of these important nutrients. Moreover, the involvement of endopeptidases belonging to serine proteases, which have an optimum pH value in the alkaline range, fits with the pH values recorded in this region of the midgut. Apparently, endopeptidases able to work at acidic or very acidic pH, such as cysteine and aspartic proteases, whose involvement in protein digestion has been demonstrated in other insects, including brachycerous larvae (Espinoza-Fuentes and Terra, 1987; Lemos and Terra, 1991b; Terra and Ferreira, 1994; Padilha et al., 2009; Fialho et al., 2012), play only a marginal role in *H. illucens* larvae as the total proteolytic activity measured in the anterior and middle regions of the midgut is negligible compared to the posterior midgut. In this region serine proteases are active and therefore represent the major enzymes responsible for the initial phase of protein digestion.

We have also unraveled the influence of temperature on proteolytic activity in the midgut of BSF larvae. In accordance with previous findings (Kim et al., 2011b), we observed an optimum temperature of around 45°C. This result is not surprising if we consider that BSF larvae exhibit a very peculiar tendency to aggregate into the feeding substrate, forming clusters in which the temperature increases due to the larval overcrowding and the heat generated by their movement (Parra Paz et al., 2015). In our rearing conditions (environmental temperature of 27.0 ± 0.5°C), a temperature higher than 40°C was recorded within the clusters, therefore proteolytic enzymes can work at their optimum temperature. This feature can strongly contribute to the efficiency of BSF larvae in the bioconversion of feeding substrates.

## Conclusion

**FIGURE 14:**
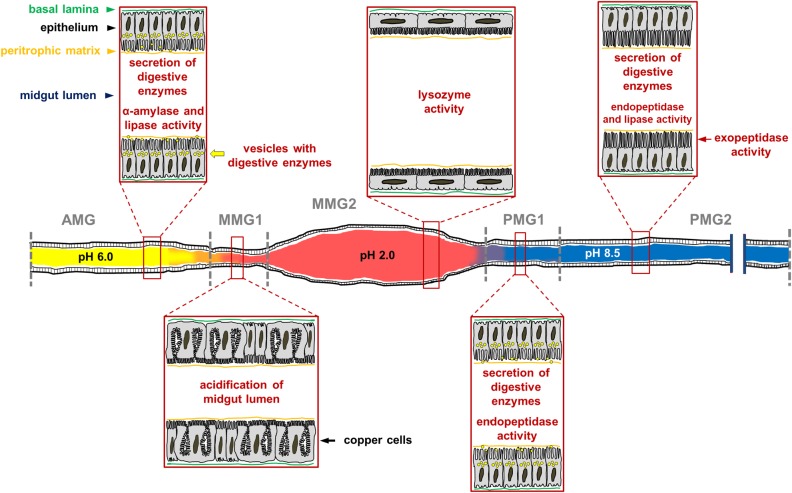
Schematic representation of *H. illucens* larval midgut in which the main morphofunctional features of each region of the organ are reported. The luminal pH of the three regions is very different, being acidic, strongly acidic, and alkaline in the anterior, middle, and posterior midgut, respectively. The AMG is characterized by columnar cells with secretory activity; in this region ingested polysaccharides begin to be degraded by soluble amylases and lipids are hydrolyzed by lipase. Copper cells, which are responsible for the highly acidic pH in the luminal content of the middle midgut, are localized in the first tract of the middle midgut (MMG1). The epithelium of the second part of the middle midgut (MMG2) is formed by large, flat cells; in this tract digestive processes do not occur, but the high activity of lysozyme and the strong luminal pH indicate an important role of this region in killing ingested pathogens. The posterior region of the midgut plays a fundamental role in protein digestion thanks to endo-and eso-peptidases, and it is responsible for further digestion of lipids. The first tract of this region (PMG1) is characterized by columnar cells with secretory activity, while the second one (PMG2) presents columnar cells with microvilli that are longer than in other regions, suggesting a main role of these cells in nutrient absorption.

Our work represents the first comprehensive description of the morphofunctional features of *H. illucens* larval midgut and sheds light on the unexpected complexity of this organ (Figure 14). Moreover, it also represents a useful platform of knowledge for best exploit this insect in bioconversion processes.

## Author Contributions

MC, GT, and SCac designed the research. MB, DB, and GS performed the research. MB, DB, MC, GT, SCap, and CJ analyzed the data. MC, SCac, and GT wrote the paper.

## Conflict of Interest Statement

The authors declare that the research was conducted in the absence of any commercial or financial relationships that could be construed as a potential conflict of interest.

## Footnotes

1 https://ilkkataponen.com/entomology-company-database/
