# Supplementary figures and images for: Structural and Functional Characterization of Hermetia illucens Larval Midgut
Source: Front Physiol. 2019 Mar 8;10:204. doi: 10.3389/fphys.2019.00204 (PMC6418021; doi:10.3389/fphys.2019.00204)

**Figure S1.** Western blot analysis: full lane of phospho-Histone 3.

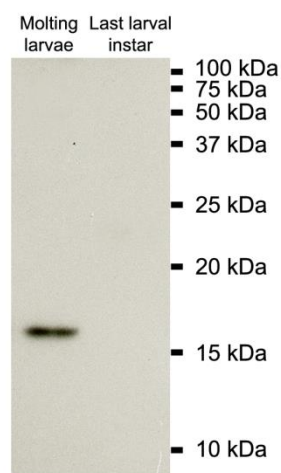

Supplement: Supplementary file 1 [file Data_Sheet_1.PDF]
