# Supplementary material for: Structural and Functional Characterization of Hermetia illucens Larval Midgut
Source: Front Physiol. 2019 Mar 8;10:204. doi: 10.3389/fphys.2019.00204 (PMC6418021; doi:10.3389/fphys.2019.00204)
Supplement: Supplementary file 2 [file Data_Sheet_2.PDF]

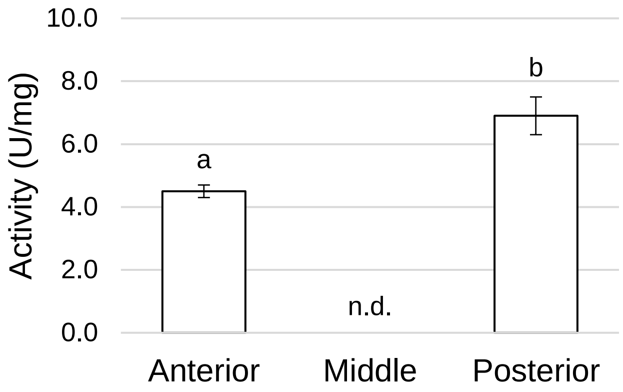

Figure S2. Lipase activity in the different midgut regions. The values are reported as mean ± SEM of at least 4 experiments. In the middle midgut no activity was detectable (n.d.). Different letters denote significant differences (unpaired *t*-test: p-value<0.05).
